# Combined Analysis of Transcriptomes and Metabolomes Reveals Key Genes and Substances That Affect the Formation of a Multi-Species Biofilm by Nine Gut Bacteria

**DOI:** 10.3390/microorganisms13020234

**Published:** 2025-01-22

**Authors:** Ting Zhang, Zhangming Pei, Hongchao Wang, Jianxin Zhao, Wei Chen, Wenwei Lu

**Affiliations:** 1 State Key Laboratory of Food Science and Technology, Jiangnan University, Wuxi 214122, China; 2 School of Food Science and Technology, Jiangnan University, Wuxi 214122, China; 3 National Engineering Research Center for Functional Food, Jiangnan University, Wuxi 214122, China

**Keywords:** M9, multi-species biofilm, gut bacteria, transcriptome, metabolome

## Abstract

Biofilms are one of the ways microorganisms exist in natural environments. In recent years, research has gradually shifted its focus to exploring the complexity and interactions of multi-species biofilms. A study showed that nine gut bacteria can form a multi-species biofilm on wheat fibers (M9 biofilm). However, the previous study did not clarify the reasons why M9 exhibited a better biofilm formation ability than the mono-species biofilms. In this study, the gene expression levels and metabolic accumulation of the M9 multi-species biofilm and biofilms of each individual bacterium were analyzed using transcriptomes and metabolomes. The differentially expressed genes (DEGs) showed that there were 740 common DEGs that existed in all of the nine groups, and they could regulate five pathways related to bacterial motility, cellular communication, and signal transduction. The metabolome results revealed that many peptides/amino acids and derivatives were produced in the M9 biofilm. Furthermore, purine metabolism was significantly enhanced in the M9 biofilm. L-arginine, l-serine, guanosine, and hypoxanthine were the common differentially accumulated metabolites (DAMs). The combined analysis of the transcriptomes and metabolomes showed that there were 26 common DEGs highly correlated with the four common DAMs, and they were involved in five metabolic pathways related to amino acids and purines. These results indicate that M9 can regulate multi-species biofilm formation by modulating genes related to bacterial motility, cellular communication, signal transduction, and the metabolism of amino acids and purines. This study provides insights into the interactions of microbial biofilms.

## 1. Introduction

Biofilms refer to an aggregation status on biological or non-biological surfaces [1]. There are significant differences between bacteria in biofilms and planktonic bacteria in terms of metabolic capacity, growth rate, gene expression, life behavior, and structural appearance [2]. These observations mainly come from studies of mono-species biofilms. However, biofilms in natural environments are usually composed of multiple microorganisms. Researchers have already found that multi-species biofilms can endow strains with certain evolutionary and unique functions that do not exist in mono-species biofilms [3]. The interactions in bacterial communities can typically alter the structure and function of the entire biofilm community [4]. Therefore, in recent years, studies of biofilms have gradually shifted to researching the complexity and interactions of multi-species biofilms [5]. But the complex composition of bacteria makes it difficult to analyze the formation mechanism and bacterial interactions of multi-species biofilms.

With the development of bioinformatics technology, omics technologies can provide comprehensive information on bacteria, genes, mRNA, metabolites, and protein expression and regulation to analyze the complex bacterial interactions of multi-species biofilms [6,7]. For example, global transcriptional analysis of *Candida albicans* and *Staphylococcus* aureus multi-species biofilms found that *C. albicans* induced the activation of an *S. aureus* biofilm formation network by downregulating the *lrg* operon, an autolysis inhibitor, and upregulating the *ica* operon, which increased the production of eDNA. The increased amount of eDNA enhanced the resistance of *S. aureus* to vancomycin [8]. *Bifidobacterium longum*, *Enterococcus faecalis*, *Bacteroides ovatus*, and *Lactobacillus gasseri* formed a multi-species biofilm and showed higher biofilm production than the mono-species biofilms. Metabolome analysis revealed that amino acids and purines promoted the formation of a multi-species biofilm. The transcriptomic results indicated that many genes related to carbohydrate metabolism, amino acid metabolism, and the environmental tolerance of *Bifidobacterium longum* were upregulated in the multi-species biofilm. Combined analysis of the metabolome and quorum-sensing mechanism found that the metabolism of proline, glycerol-leucine, N-(3-oxohydroxy) homoserine lactones, and autoinducer 2 (AI-2) promoted the formation of a multi-species biofilm [2]. Thus, omics technologies are crucial for analyzing the formation of multi-species biofilms, key biomarkers, and interaction mechanisms.

The intestine is the organ with the largest number and variety of microorganisms. Observations of the geographical distribution of gut microbiota reveal that biofilms grow throughout the gastrointestinal tract. In addition to the outer mucus layers and gut epithelium surfaces, undigested food particles are also one of the important microbial aggregation niches [9]. Undigested food particles commonly come from insoluble dietary fibers ingested in the daily diet. A study showed that 33 types of bacteria were found to adhere to undigested food particles by isolating and sequencing 15 healthy donors’ food residues from their feces [10]. However, few studies use undigested food particles as carriers to reconstruct biofilms in vitro. Ushakova et al. showed that *Lactobacillus plantarum* 8R-A3 can form a mono-species biofilm on wheat bran within 48 h, and this biofilm is beneficial for *Lactobacilli* cell survival during the drying of the fermentation mass [11]. Liu et al. explored the mono-species biofilm formation ability of six gut bacteria (*Bifidobacterium animalis*, *Bifidobacterium bifidum*, *Bifidobacterium adolescentis*, *Bifidobacterium pseudo*, *Bifibacterium breve*, and *Bifidobacterium longum*) in grape seed flour [12]. In addition, *Bifidobacterium bifidum*, *Bifidobacterium pseudo*, and *Bifidobacterium longum* can also form mono-species biofilms on wheat fibers with particle sizes of around 50 μm [6,13,14].

In a previous study, our group successfully constructed a multi-species biofilm on wheat fibers formed by nine core gut bacteria (*Bacteroides ovatus*, *Parabacteroides distasonis*, *Bacteroides uniformis*, *Bacteroides cellulosilyticus*, *Escherichia coli*, *Bacteroides fragilis*, *Phocaeicola dorei*, *Bacteroides stercoris*, and *Bifidobacterium longum*), called the M9 biofilm [15]. This multi-species biofilm showed the best biofilm formation ability compared to each mono-species biofilm and formed tight structures on wheat fibers. Furthermore, M9 retained a better biofilm formation ability under pH and bile salt stresses. However, our previous study did not clearly identify why M9 exhibited a better biofilm formation ability than the mono-species biofilms. Based on this, this study aims to identify key genes and metabolites that affect M9 biofilm formation using transcriptomes and metabolomes. This study is one of the few reports focusing on the formation mechanism of a multi-species biofilm composed of gut bacteria. The findings of this study can provide insights into the research of muti-species biofilms, especially using insoluble dietary fibers as carriers.

## 2. Materials and Methods

### 2.1. Biofilm Formation

*Bacteroides ovatus* CCFM1342, *Parabacteroides distasonis* CCFM1377, *Bacteroides uniformis* CCFM1358, *Bacteroides cellulosilyticus* FSDTAELIBHI5, *Escherichia coli* CCFM1378, *Bacteroides fragilis* FSDTAHCK_B8, *Phocaeicola dorei* FSDLZ62K4, *Bacteroides stercoris* FFJLY21K3, and *Bifidobacterium longum* FBJCY2M11 were used in this study. All strains were obtained from Jiangnan University (Wuxi, China).

The nine bacteria were activated using yeast extract, casitone, and fatty acid liquid media (YCFA liquid media; Table S1) at 37 °C in an anaerobic incubator (Electrotek Inc., West Yorkshire, UK). To obtain mono-species and M9 biofilms, wheat fibers with particle sizes around 45 μm were employed as carriers. Mono-species and M9 biofilms were prepared using the dynamic fermentation method described in the previous study. Briefly, each activated strain was diluted to an absorbance value of 0.1 at 600 nm (10^7^–10^8^ CFU per mL) using fresh YCFA liquid media. Each diluted mono-species solution was mixed in equal volumes to obtain the M9 multi-species solution. A 2% (*v*/*v*) diluted mono-species or M9 multi-species solution was added to the dynamic fermentation medium with 4% (*w*/*v*) wheat fibers. The dynamic fermentation media were anaerobically cultivated in a constant temperature shaking incubator ( Zhichu Instrument Co., Ltd., Shanghai, China) at 120 rpm and 37 °C for 24 h [15].

### 2.2. RNA Extraction and Sequencing

Mono-species and M9 biofilms prepared using the dynamic fermentation method were placed in RNase-free centrifuge tubes. Samples were centrifuged at 100 rpm for 2 min. The precipitates were biofilms, and the upper liquids were planktonic bacteria. Biofilm samples were washed twice using PBS to remove suspended cells. The cell count of each biofilm sample was >10^8^ CFU per mL. Biofilm samples were rapidly frozen in liquid nitrogen and then stored at −80 °C for RAN extraction.

Total RNA was extracted from mono-species and M9 biofilms using an RNAprep Pure Cell/Bacteria Kit (content number: DP430; Tiangen Biotech Co., Ltd., Beijing, China). The concentration and integrity of the RNA were determined using a NanoDrop (Thermo Fisher Scientific Inc., Waltham, MA, USA) and Agilent 2100 (Agilent Technologies Inc., Santa Clara, CA, USA). The purity of RNA was detected by agarose gel electrophoresis using an Agilent 2100 [16]. The library construction and sequencing were performed by Novogene (Novogene Bioinformatics Technology Co., Ltd., Beijing, China). The library quality was assessed using the Agilent 5400 system (Agilent Technologies Inc., Santa Clara, CA, USA) and quantified using QPCR (1.5 nM). The qualified library was pooled and sequenced on the Illumina platform using the PE150 strategy in Novogene. Raw data were obtained by transforming the original fluorescence image files to short reads through base calling. Fastp (version 0.23.1) was used to perform basic statistics on the quality of raw reads and to obtain clean data [17]. Q20 (recognition error rate < 1%) and Q30 (recognition error rate < 0.1%) were used to evaluate the quality of sequenced bases.

### 2.3. Transcriptome Analysis

Clean data were used for subsequent analyses. Reference genomes for the nine bacteria were downloaded from the National Center for Biotechnology Information (NCBI) to map clean reads through Spliced Transcripts Alignment to a Reference (STAR) software (vision 2.7.10b) [18]. Mapped reads were counted using featureCounts (vision 2.0.3) [19]. The DEGs of the nine comparison groups (M9 vs. *B. ovatus*, M9 vs. *P. distasonis*, M9 vs. *B. uniformis*, M9 vs. *B. cellulosilyticus*, M9 vs. *E. coli*, M9 vs. *B. fragilis*, M9 vs. *P. dorei*, M9 vs. *B. stercoris*, and M9 vs. *B. longum*) were obtained by edgeR (version 4.2.1, R package) [20] assuming *p* < 0.05 and |log_2_ (fold change)| > 1 [21]. Common DEGs shared by the nine groups were analyzed using UpSetR (version 1.4.0, R package). ClusterProfiler (version 4.12.6, R package) was used to analyze the Kyoto Encyclopedia of Genes and Genomes (KEGG) pathways of DEGs [22]. The data presented were deposited in the NCBI repository under accession number: PRJNA1188810.

### 2.4. Metabolite Extraction and Determination

Biofilm samples were centrifuged at 14,000 rpm for 5 min at 4 °C. A 100 μL supernatant was mixed with a 400 μL aliquot of 1:1 (*v*/*v*) ice-cold mixture of methanol and acetonitrile, and ultrasound was performed with an ice bath for 10 min. Then, the samples were stored at −20 °C for 1 h to precipitate cellular debris and protein. Subsequently, the samples were centrifuged at 14,000 rpm for 15 min at 4 °C. The supernatants were transferred to new round-bottomed centrifuge tubes and dried for 4 h in a vacuum concentrator. Each dried sample was resolved in 200 μL 50% (*v*/*v*) acetonitrile solution and vortexed for 30 s. A 100 μL aliquot of metabolite extraction was injected into an autosampler vial and stored at −80 °C until further use [15]. Each group comprised six biological replicates.

The metabolite extracts were determined by ultra-performance liquid chromatography–mass spectrometry (UHPLC-MS) [23]. A Kinetex C18 column (2.1 × 100 mm, 2.6 μm, Phenomenex Inc., Torrance, CA, USA) was used for the separation of metabolites at 35 °C. The sample injection volume was 2 μL at a flow rate of 0.3 mL per min. Mobile phase A consisted of 0.01% acetic acid and water, whereas mobile phase B consisted of 50% acetonitrile and 50% isopropanol. The elution gradient (A:B, *v*/*v*) was 80:20 from 0 to 1 min, 0:100 at 7 min, maintained for 4 min, and then 80:20 at 11 min, maintained for 2 min. Data-dependent acquisition was used for the tandem MS workflow in both the positive and negative ion modes [15].

### 2.5. Untargeted Metabolome Data Analysis

Untargeted metabolome data were used to analyze the metabolic profiles of the mono- and M9 biofilms. Raw data files were analyzed using Compound Discoverer (version 3.3) for annotation and putative identification of metabolites. The DAMs were analyzed using MetaboAnalyst 6.0 (https://www.metaboanalyst.ca/, accessed on 25 July 2024) [2], including M9 vs. *B. ovatus*, M9 vs. *P. distasonis*, M9 vs. *B. uniformis*, M9 vs. *B. cellulosilyticus*, M9 vs. *E.coli*, M9 vs. *B. fragilis*, M9 vs. *P. dorei*, M9 vs. *B. stercoris*, and M9 vs. *B. longum* groups. The common DAMs shared by the nine groups were analyzed using UpSetR. The DAMs were input into MetaboAnalyst 6.0 to obtain KEGG metabolic pathways.

### 2.6. RT-qPCR

RT-qPCR was used to verify the expression levels of the concerned genes. Total RNA from the mono- and M9 biofilms was extracted using a Bacteria RNA Extraction Kit (R403; Vazyme Biotech Co., Ltd., Nanjing, China). The quality and concentration of total RNA were evaluated using a Nanophotometer N60 Touch (IMPLEN, Munich, Germany). cDNA was synthesized from total RNA (1 μg) using HiScript IV All-in-One Ultra RT SuperMix for qPCR (R433; Vazyme Biotech Co., Ltd., Nanjing, China). The final qPCR system was 10 μL: 1 μL cDNA template, 0.2 μL forward primer, 0.2 μL reverse primer, 3.6 μL ddH_2_O, and 5 μL SYBR Green Premix Ex Taq II. The RT-qPCR program was as follows: 95 °C, 2 min s; 40 cycles of 95 °C, 10 s; and 60 °C, 30 s. The melt curve was 65 °C to 95 °C, 0.5 °C per 5 s. The expression levels of these 14 genes were normalized by a reference gene designed in this study. The messages of specific primers are listed in Table 1. The relative expression levels were calculated by the 2^−ΔΔCt^ method.

### 2.7. Statistical Analysis

In addition to the data analysis methods described above, volcano plots, KEGG enrichment plots, heatmaps, correlation heatmaps, and bubble plots were visualized using ChiPlot (https://www.chiplot.online/, accessed on 29 July 2024). Correlation net plots of DAMs and DEGs were visualized using Gephi (version 0.10.1). Column plots and point-and-figure plots were performed and visualized using Origin (version 2017).

## 3. Results

### 3.1. Global Transcription Levels of Mono-Species and M9 Biofilms

**Figure 1:**
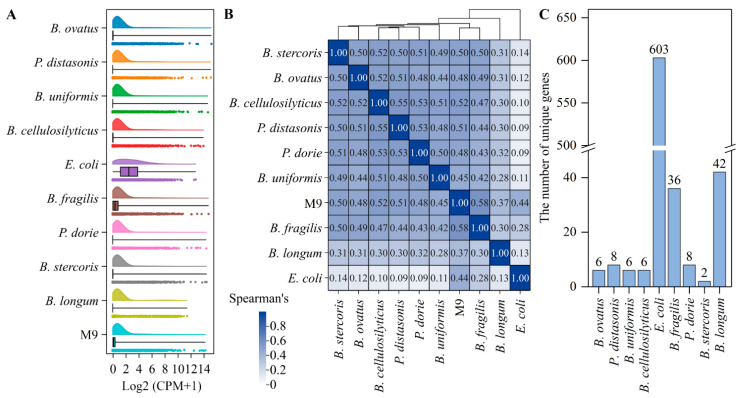
Global transcription levels of mono-species and M9 biofilms. (**A**) The raincloud plot of gene expression levels for mono-species and M9 biofilms. (**B**) Spearman’s correlations of gene expression levels of mono-species and M9 biofilms. (**C**) The number of unique genes of each mono-species biofilm.

Transcriptome analysis was used to distinguish differences in the gene expression levels of each strain and M9 after biofilm formation on wheat fibers. Transcriptome sequencing totally obtained 39.42 GB of clean data. The number of bases with a recognition error rate ≤ 1% (Q20) in each sample was ≥96.61%, and those with a recognition error rate ≤ 0.1% (Q30) were ≥91.25% (Table 2). The distribution of the RNA-seq data was concentrated and contained a few outliers (Figure 1A). The nine bacterial strains used in this article belong to three different phyla: *Bacteroidetes* (*B. ovatus*, *P. distasonis*, *B. uniformis*, *B. cellulosilyticus*, *B. fragilis*, *P. dorei*, and *B. stercoris*), *Proteobacteria* (*E. coli*), and *Actinobacteria* (*B. longum*). The result of Spearman’s correlation analysis revealed that the correlations of gene expression among the *B. ovatus*, *P. distasonis*, *B. uniformis*, *B. cellulosilyticus*, *B. fragilis*, *P. dorei*, and *B. stercoris* mono-species biofilms were closer (Spearman’s correlation coefficient ≥ 0.42), while the gene expression of these mono-species biofilms had lower correlations with the *E. coli* and *B. longum* biofilms (Spearman’s correlation coefficient ≤ 0.32). The *E. coli* biofilm had the lowest Spearman’s correlation with the other mono-species biofilms (Figure 1B). The *B. ovatus*, *P. distasonis*, *B. uniformis*, *B. cellulosilyticus*, *E. coli*, *B. fragilis*, *P. dorei*, *B. stercoris*, *B. longum*, and M9 biofilms had 6, 8, 6, 6, 603, 36, 8, 2, 42, and 10 unique genes (Figure 1C). The transcriptome analysis confirmed that the qualities of RNA-seq data were high, and the samples were uncontaminated [2]. Furthermore, the correlations of gene expression among the *Bacteroidetes* phylum were higher, but they had lower correlations with *E. coli* and *B. longum*. This might be because the seven bacterial strains belonging to the *Bacteroidetes* phylum have more similar genomes than *E. coli* and *B. longum* and therefore exhibited more similar gene expression in the biofilms [24].

### 3.2. The DEGs and Function Analysis of Mono-Species and M9 Biofilms

**Figure 2:**
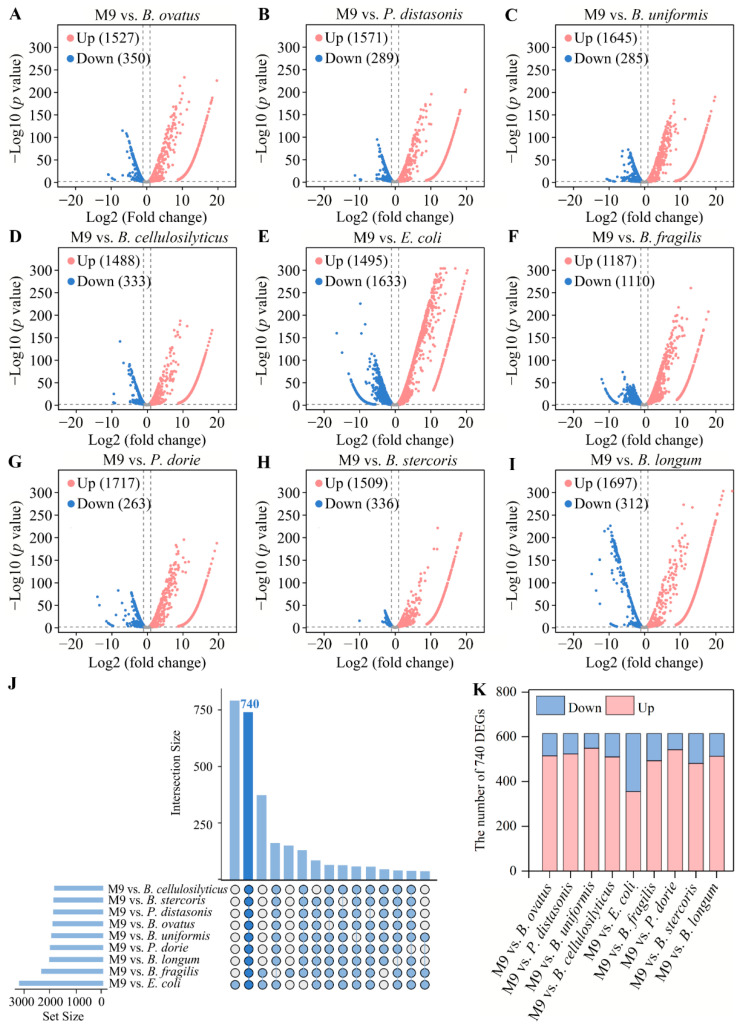
The DEGs analysis. (**A**–**I**) The volcano plots of the M9 vs. *B. ovatus*, M9 vs. *P. distasonis*, M9 vs. *B. uniformis*, M9 vs. *B. cellulosilyticus*, M9 vs. *E. coli*, M9 vs. *B. fragilis*, M9 vs. *P. dorei*, M9 vs. *B. stercoris*, and M9 vs. *B. longum* groups. (**J**) The Upset plot of common DEGs from nine groups. (**K**) The number of upregulated and downregulated common DEGs in each group.

To compare gene expressions among the mono-species and M9 biofilms, the M9 biofilm was used as a control to obtain DEGs. We observed the following: for the M9 vs. *B. ovatus* group, a total of 1877 DEGs, with 1527 upregulated and 350 downregulated (Figure 2A); for the M9 vs. *P. distasonis* group, a total of 1860 DEGs, with 1571 upregulated and 289 downregulated (Figure 2B); for the M9 vs. *B. uniformis* group, a total of 1930 DEGs, with 1645 upregulated and 285 downregulated (Figure 2C); for the M9 vs. *B. cellulosilyticus* group, a total of 1821 DEGs, with 1488 upregulated and 333 downregulated (Figure 2D); for the M9 vs. *E. coli* group, a total of 3128 DEGs, with 1495 upregulated and 1633 downregulated (Figure 2E); for the M9 vs. *B. fragilis* group, a total of 2297 DEGs, with 1187 upregulated and 1110 downregulated (Figure 2F); for the M9 vs. *P. dorei* group, a total of 1980 DEGs, with 1717 upregulated and 263 downregulated (Figure 2G); for M9 vs. *B. stercoris* group, a total of 1845 DEGs, with 1509 upregulated and 336 downregulated (Figure 2H); and for the M9 vs. *B. longum* group, a total of 2009 DEGs, with 1697 upregulated and 312 downregulated (Figure 2I). The DEG analysis showed that the M9 biofilm produced more significant gene expression changes compared with those of the mono-species biofilms, and the number of DEGs varied among different strains. The gene expressions of the multi-species biofilm are influenced by all strains in the mixed system and can exhibit neutral, cooperative, or competitive interactions [21,25]. These interactions may result in each strain exhibiting different gene expression patterns in multi-species biofilms compared with those of mono-species biofilms, leading to better environmental adaptability and growth and altering metabolic functions [4,26].

The functions of the DEGs were analyzed by KEGG enrichment analysis. The nine groups were enriched in the same 86 functional pathways. The top 10 KEGG pathways for each group are shown in Figure S1A–I. The 86 KEGG pathways were divided into 21 subcategories (Figure S1J). These pathways play important roles in the formation, interactions, and functional phenotypes of multi-species biofilms. Among them, the KEGG pathways related to amino acid metabolism and carbohydrate metabolism had the highest DAM number, which can effectively improve energy utilization, maintain the balance of the redox state, adapt to various environmental conditions, and offer sufficient nutritional availability. In addition, pathways related to bacterial motility, cellular communication, and signal transduction were also included in the 86 KEGG pathways, which not only provide more opportunities for bacteria to adhere to carriers but also facilitate interactions at the mature stage. These results indicated that the M9 biofilm had more complex gene expression patterns than each mono-species biofilm. Many function pathways related to bacterial growth, substance metabolism, and signal transduction were significantly activated in the multi-species environment.

### 3.3. Common DEG Analysis and Validation

**Figure 3:**
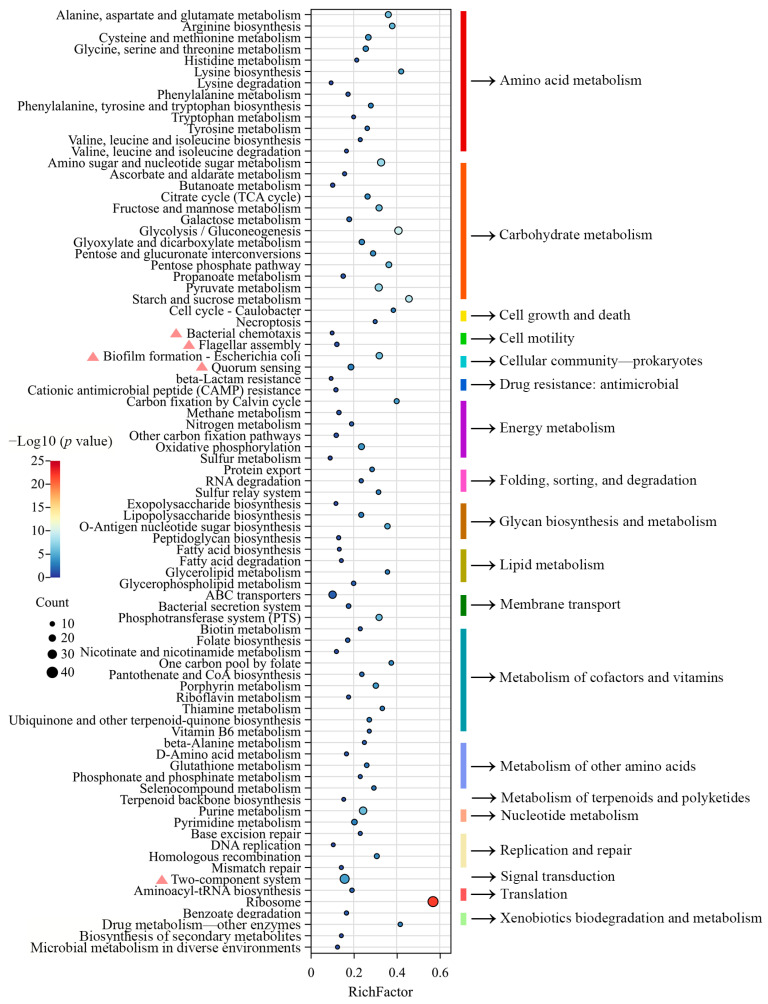
The KEGG enrichment analysis of 740 common DEGs. Pink triangles indicate KEGG pathways related to bacterial motility, cellular communication, and signal transduction.

**Figure 4:**
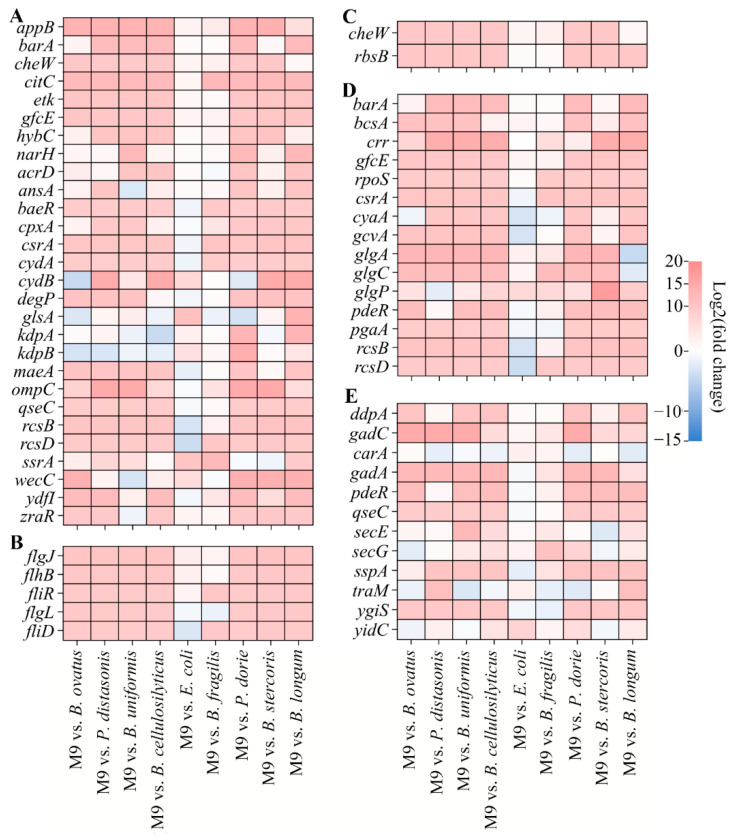
Heatmaps of common DEGs related to bacterial motility, cellular communication, and signal transduction. (**A**) Two-component system pathway. (**B**) Flagellar assembly pathway. (**C**) Bacterial chemotaxis pathway. (**D**) Biofilm formation—*Escherichia coli* pathway. (**E**) Quorum sensing pathway.

The 86 identical KEGG pathways indicated that the mixing of the nine bacteria might regulate some genes with similar functions. By taking the intersection of DEGs among all groups, it was found that 740 DEGs were shared among the nine groups (Figure 2J). The number of upregulated common DEGs was higher than that of downregulated common DEGs (Figure 2K). The 740 common DEGs were involved in 83 functional pathways, which were divided into 20 subcategories, including amino acid metabolism (13), carbohydrate metabolism (13), cell growth and death (2), cell motility (2), cellular community-prokaryotes (2), drug resistance: antimicrobial (2), energy metabolism (6), folding, sorting, and degradation (3), glycan biosynthesis and metabolism (4), lipid metabolism (4), membrane transport (3), metabolism of cofactors and vitamins (10), metabolism of other amino acids (5), metabolism of terpenoids and polyketides (1), nucleotide metabolism (2), replication and repair (4), signal transduction (1), translation (2), xenobiotics biodegradation and metabolism (2), and unknown (2) (Figure 3). In addition, the result showed that there were 28, 20, 15, 12, 2, and 5 common DEGs involved in the two-component system, biofilm formation—*Escherichia coli*, quorum sensing, bacterial chemotaxis, and flagellar assembly (Figure 4). The five functional pathways are considered to be related to bacterial motility, cellular communication, and signal transduction, which might be regulated by mixed bacterial behavior.

**Figure 5:**
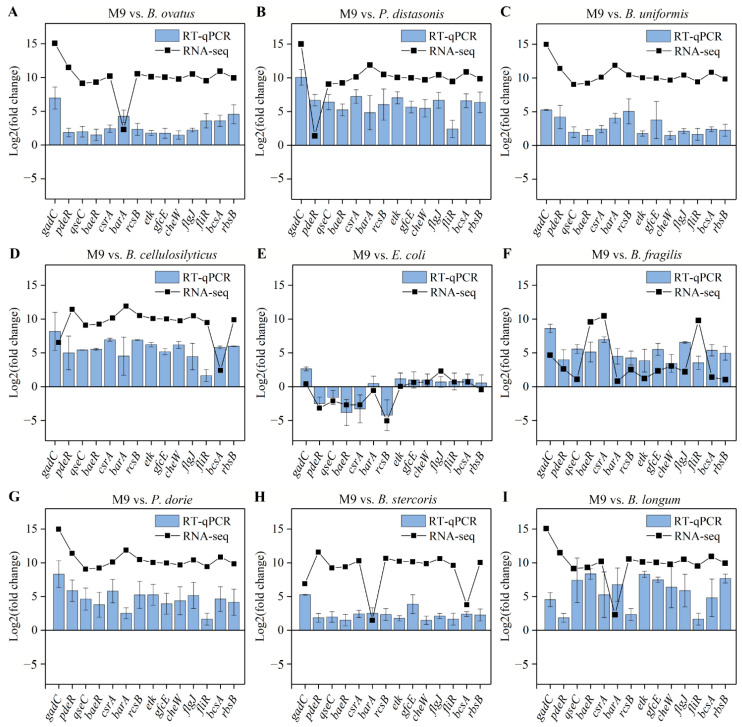
The RT-qPCR validation of 14 common DEGs in the M9 vs. *B. ovatus* M9 vs. *P. distasonis*, M9 vs. *B. uniformis*, M9 vs. *B. cellulosilyticus*, M9 vs. *E. coli*, M9 vs. *B. fragilis*, M9 vs. *P. dorei*, M9 vs. *B. stercoris*, and M9 vs. *B. longum* groups (**A**–**I**).

To affirm the accuracy and reliability of the RNA-seq data, 14 common DEGs from the five KEGG pathways were validated by RT-qPCR using specific primers (Table 1), including *gadC*, *pdeR*, *qseC*, *baeR*, *csrA*, *barA*, *rcsB*, *etk*, *gfcE*, *cheW*, *flgJ*, *fliR*, *bcsA*, and *rbsB*. Comparative analysis showed that the RT-qPCR expression profiles of all the common DEGs were in agreement with the RNA-seq analysis in the nine groups (Figure 5). The verification results demonstrated that RNA-seq data had high accuracy.

### 3.4. Untargeted Metabolome Analysis of Mono-Species and M9 Biofilms

**Figure 6:**
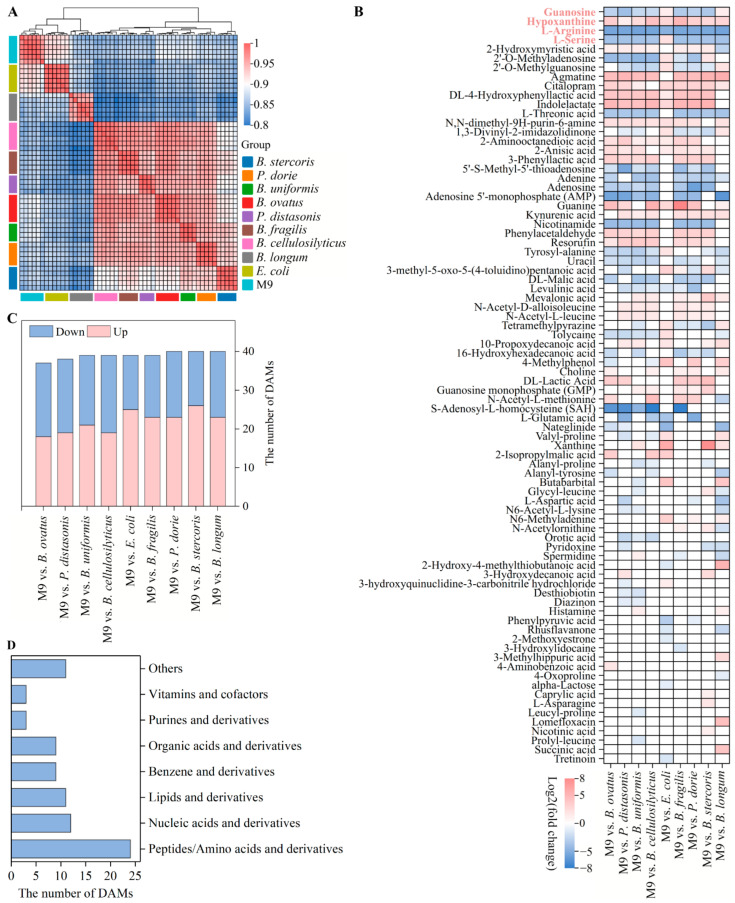
Untargeted metabolome analysis of mono-species and M9 biofilms. (**A**) Spearman correlation of metabolites among the nine groups. (**B**) The 82 DAMs identified in the nine groups. The legend indicates log2 (fold change) of mono-species and M9 biofilms. Pink words indicate four common DAMs. (**C**) The number of upregulated and downregulated DAMs of each group. (**D**) The classification of the 82 DAMs.

**Figure 7:**
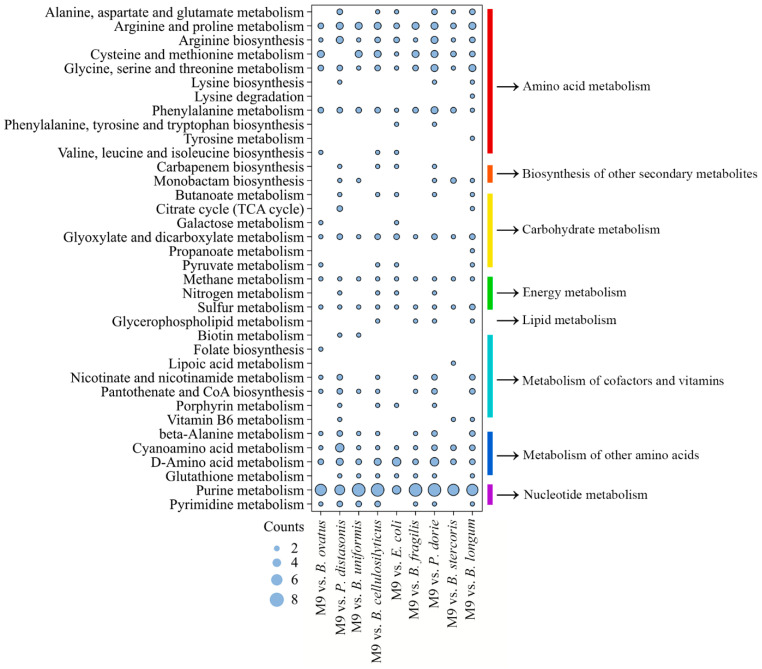
The KEGG enrichment analysis of the DAMs from each group.

The metabolites of the mono-species and M9 biofilms were detected using UHPLC-MS. The metabolites of the M9 and *E. coli* biofilms had the highest positive correlation (Spearman’s correlation coefficient ≥ 0.88; Figure 6A). According to the threshold, there were a total of 82 DAMs (Figure 6B), wherein 37, 38, 39, 39, 39, 39, 40, 40, and 40 DAMs were screened in the M9 vs. *B. ovatus* group (18 upregulated, 19 downregulated), the M9 vs. *P. distasonis* group (19 upregulated, 19 downregulated), the M9 vs. *B. uniformis* group (21 upregulated, 18 downregulated), the M9 vs. *B. cellulosilyticus* group (19 upregulated, 19 downregulated), the M9 vs. *E. coli* (25 upregulated, 14 downregulated), the M9 vs. *B. fragilis* group (23 upregulated, 16 downregulated), the M9 vs. *P. dorei* group (23 upregulated, 17 downregulated), the M9 vs. *B. stercoris* group (23 upregulated, 14 downregulated), and the M9 vs. *B. longum* group (23 upregulated, 17 downregulated), respectively (Figure 6C). L-arginine, l-serine, guanosine, and hypoxanthine were the common DAMs found in the nine groups. A total of 24, 12, 11, 9, 9, 3, 3, and 11 DAMs belonged to peptides/amino acids and derivatives, nucleic acids and derivatives, lipids and derivatives, benzene and derivatives, organic acids and derivatives, purines and derivatives, vitamins and cofactors, and others, respectively (Figure 6D). The 82 DAMs were enriched in 36 different metabolic pathways, which were classified as amino acid metabolism (11), biosynthesis of other secondary metabolites (2), carbohydrate metabolism (6), energy metabolism (3), lipid metabolism (1), metabolism of cofactors and vitamins (7), metabolism of other amino acids (4), and nucleotide metabolism (2). The DAMs of the M9 vs. *B. ovatus*, M9 vs. *P. distasonis*, M9 vs. *B. uniformis*, M9 vs. *B. cellulosilyticus*, M9 vs. *E. coli*, M9 vs. *B. fragilis*, M9 vs. *P. dorei*, M9 vs. *B. stercoris*, and M9 vs. *B. longum* groups were enriched in 19, 25, 18, 24, 21, 17, 25, 15, and 27 KEGG metabolic pathways, respectively (Figure 7). The top 10 KEGG pathways of each group are shown in Figure S2A–J. These results revealed that many amino acids were produced in the M9 biofilm compared with those in the mono-species biofilms, and many amino acid metabolic pathways were affected. In addition, purine metabolism was most significantly affected by the M9 multi-species biofilm.

### 3.5. Combined Analysis of Metabolomes and Transcriptomes

**Figure 8:**
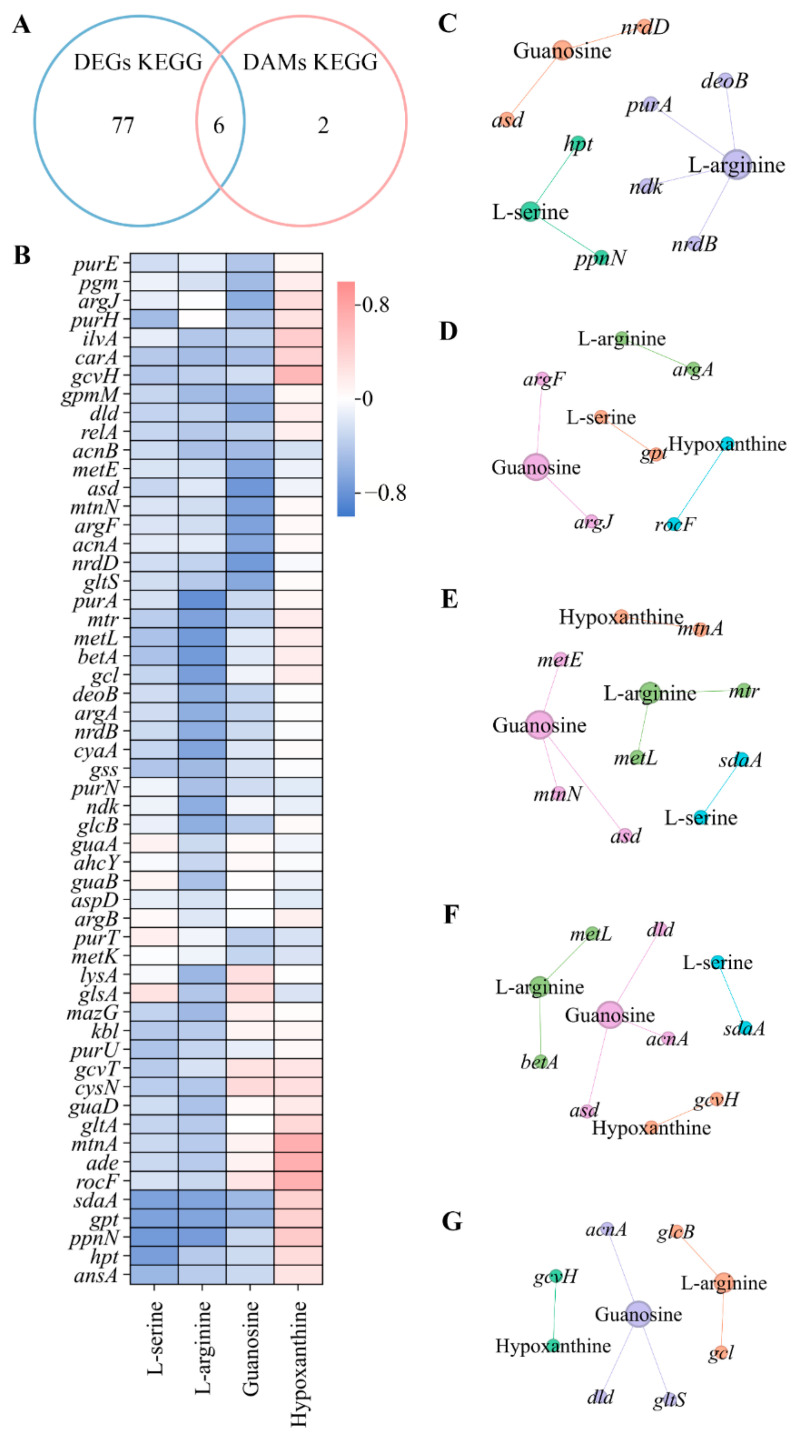
Combined analysis of common DEGs and common DAMs. (**A**) The Venn plot of KEGG pathways between 740 common DEGs and four common DAMs. (**B**) Spearman’s correlations of common DEGs and common DAMs involved in six coenriched metabolic pathways. (**C**–**G**) The correlation network of common DEGs and common DAMs in purine metabolism, arginine biosynthesis, cysteine and methionine metabolism, glycine, serine, and threonine metabolism, and glyoxylate and dicarboxylate metabolism.

The analysis of genes and metabolites revealed that there were 740 common DEGs (Figure 2J) and four common DAMs (Figure 6B), which were believed to be regulated by mixed bacterial behavior. The conjoint analysis of these common DEGs and DAMs showed that six KEGG pathways were coenriched (Figure 8A). The cor program in R was used to analyze the correlation between the DEGs and DAMs involved in the six pathways, and the results are shown in Figure 8B. To identify highly correlated DEGs and DAMs in each pathway, the screening criteria were set to the absolute value of Spearman’s correlation coefficient ≥ 0.6 and *p* < 0.05. The highly correlated common DEGs and common DAMs were involved in five pathways related to amino acid and purine metabolisms. In the purine metabolism pathway, *asd*, *deoB*, *hpt*, *ndk*, *nrdB*, *nrdD*, *ppnN*, and *purA* were negatively correlated with guanosine, l-arginine, and l-serine (Figure 8C). *argA*, *argF*, *argJ*, and *gpt* were negatively correlated with guanosine, l-arginine, and l-serine in the arginine biosynthesis pathway, while *rocF* was positively correlated with hypoxanthine (Figure 8D). In cysteine and methionine metabolism, *asd*, *metE*, *metL*, *mtnN*, *mtr*, and *sdaA* were negatively correlated with guanosine, l-arginine, and l-serine, while *mtnA* was positively correlated with hypoxanthine (Figure 8E). *gcvH* was positively correlated with hypoxanthine in the glycine, serine, and threonine metabolism pathway, while *acnA*, *asd*, *betA*, and *dld* were positively correlated with the other three DAMs (Figure 8F). In the glyoxylate and dicarboxylate metabolism pathway, *acnA*, *gcl*, *dld*, *glcB*, and *gltS* were negatively correlated with guanosine and l-arginine, while *gcvH* was positively correlated with hypoxanthine (Figure 8G). Amino acid metabolism and purine metabolism were significantly regulated by the nine mixed bacteria.

## 4. Discussion

Insoluble dietary fiber is a proven biofilm formation carrier. *B. animalis*, *B. bifidum*, *B. adolescentis*, *B. pseudo*, *B. breve*, and *B. longum* successfully formed mono-species biofilms on grape seed flour or wheat fibers through dynamic fermentation. However, microorganisms in natural environments prefer to coexist in multi-species biofilms. Metabolic exchange, nutrient utilization, and signal transduction cause microorganisms in multi-species biofilms to exhibit better biofilm formation ability or inhibit unfavorable biofilm formation. Nine gut core bacteria have been shown to form a multi-species biofilm (the M9 biofilm) on wheat fibers. The M9 biofilm showed better biofilm formation and resistance in adverse environments compared with mono-species biofilms. However, the previous study did not elucidate the reasons why M9 exhibited a better biofilm formation effect on wheat fibers than on mono-species biofilms. This study utilized transcriptomes and metabolomes to identify DEGs and DAMs between the M9 multi-species biofilm and mono-species biofilms. The DEGs of the nine comparison groups were involved in the same 86 KEGG pathways. The DAMs of the nine comparison groups were significantly involved in the purine metabolism pathway, and the greatest number of pathways belonged to amino acid metabolism. There were 740 DEGs and four DAMs shared by all nine groups, which were considered as key genes and substances influenced by M9. The 83 different functional pathways were enriched by 740 common DEGs, wherein five pathways were related to bacterial motility and cellular communication. The 740 common DEGs and four common DAMs jointly regulated six metabolic pathways, which were associated with amino acid and purine metabolisms. In addition, the combined analysis of transcriptomes and metabolomes showed that 26 common DEGs were highly correlated with four common DAMs.

Biofilm formation is a dynamic process, and the first stage is attachments between bacteria and carriers by cell poles or flagella [1]. The gene expression level of several common DEGs (*flgJ*, *flgL*, *flhB*, *fliR*, and *fliD*) related to flagella assembly was regulated by M9. Flagella are a special motility structure of bacteria composed of ~30 different flagellar proteins [27]. Compared with non-motile bacteria, motile bacteria usually show better diffusion ability, providing them more opportunities to attach to carrier surfaces and increase the number of cells per unit area [28]. In addition to flagella assembly, the KEGG enrichment analysis also revealed that M9 upregulated genes (*cheW* and *rbsB*) related to bacterial chemotaxis. Unlike flagella with a motility structure, chemotaxis is the ability of bacteria to migrate upwards or downwards, towards beneficial chemicals, or away from toxic substances when bacteria are stimulated by chemical signals in different environments. A large number of metabolites are released at the biofilm mature stage, which can be utilized by other bacteria in the environment, improving the interaction among bacteria.

Genes involved in cellular communication play an important role in M9 biofilm formation. Among the 740 common DEGs discovered in this study, 12 were involved in quorum sensing, including *gadA*, *qseC*, *pdeR*, *gadC*, *traM*, *ddpA*, *ygiS*, *sspA*, *secE*, *secG*, *yidC*, and *carA. gadC* and *ddpA* were significantly co-upregulated in the nine comparison groups. *gadC* is a glutamate antiporter gene that encodes the glutamate decarboxylase system, using AHLs as signaling molecules [29]. The glutamate-dependent acid resistance system is extremely powerful for bacterial protection in acidic environments [30]. The pH of the media significantly decreased during the dynamic fermentation process. Increased acidity can severely inhibit bacterial growth, thereby affecting the maturation of biofilms. This acidic protection system enables bacteria to counteract the increase in intracellular protons [31]. Knocking out *gadC* hindered the beneficial effects of the derepressed conjugative plasmid R1drd19 on the formation of *E. coli* biofilm [32]. *ddpA* is a putative D, D-dipeptide ATP-binding cassette transporter (ABC transporter). At present, the role of D, D-dipeptide ABC transporter in biofilm formation remains unclear [33,34]. Biofilm formation—*Escherichia coli* was another important cellular community pathway in this study. There were 15 common DEGs (*gfcE*, *pdeR*, *bcsA*, *csrA*, *crr*, *barA*, *rcsB*, *gcvA*, *cyaA*, *rcsD*, *pgaA*, *rpoS*, *glgP*, *glgA*, and *glgC*) involved in the cyclic adenosine 3′5′ monophosphate/cyclic adenosine 3′5′ monophosphate receptor protein (cAMP/CRP) signal pathway, and BarA/UvrY/CsrA system of biofilm formation—*Escherichia coli*. cAMP/CRP plays an auxiliary role in regulating the formation of *E. coli* biofilms and can directly activate the transcription of *csgD* and regulate curli fibers synthesis. cAMP/CRP can also positively regulate the transcription of *flhDC*, which controls approximately 50 gene clusters related to flagellum biosynthesis. Both curli fiber synthesis and flagellum biosynthesis are important to bacteria adhesion on carrier surfaces and affect biofilm formation [35]. BarA/UvrY is a two-component system, promoting cellular communication. BarA/UvrY can establish a link between cell metabolism and the post-transcriptional regulation system of carbon storage regulator (Csr). Many physiological processes, including biofilm formation, are coordinated by BarA/UvrY through the Csr system [36].

The two-component system had the highest number of common DEGs in this study, with a total of 28. They mainly involved in six families of the two-component system, including the OmpR family (*kdpA*, *kdpB*, *baeR*, *ompC*, *acrD*, *degP*, *cpxA*, and *qseC*), the NarL family (*csrA*, *barA*, *rcsB*, *rcsD*, *hyaB*, *narH*, and *ydfI*), the NtrC family (*qseC* and *zraR*), the CitB family (*maeA* and *citC*), LuxR family (*ssrA*), and other families (*gfcE*, *etk*, *glsA*, *wecC*, *ansA*, *appB*, *cydA*, and *cydB*). As discussed, the two-component system is beneficial for cellular communication. Moreover, as one of the most important signal transduction modes for bacteria, the two-component system can quickly respond to environmental changes using various mechanisms, such as cross-regulation, to integrate and coordinate various input stimuli and control biofilm formation [37]. *B. longum* could regulate the secretion of type IV pili and the extracellular matrix through the LuxC/LuxE two-component system [14]. Bbi37|peg.341, *degP*, *pstS*, and *vanY* were associated with the two-component system and could influence the formation of the *B. bifidum* biofilm on wheat fibers [38]. Genes related to biofilm formation (*epsA*, *epsB*, *epsC*, and *tasA*) were significantly downregulated when knocking out two-component system genes, i.e., *degQ* and *degU*, in the *Bacillus velezensis* strain DMW1 [27].

Amino acids were the most important substances that participated in the M9 biofilm formation (Figure 6B,D). Amino acids are important for facilitating efficient energy utilization, maintaining a balanced redox state, adapting to various environmental conditions, and providing sufficient nutrients [39]. In this study, only two amino acids (l-arginine and l-serine) were common DAMs, which indicated that their accumulation levels in the biofilms were significantly influenced by M9. L-arginine was co-downregulated in the nine groups. L-arginine can produce ammonia via the arginine deiminase system [40]. Ammonia can neutralize acids produced during the fermentation process, thereby protecting microbial aggregations from acid stress [41]. However, the stability of l-arginine on microbial aggregations relies on the concentration of l-arginine. Micromolar concentrations of l-arginine metabolism exhibit the stabilizing ability of bacterial co-aggregation and can mediate intercellular signaling in biofilms [42], while high concentrations (>50 μM) of l-arginine reduce bacterial adhesion, decreasing the formation of biofilms [43]. L-serine was significantly downregulated in the M9 vs. *B. ovatus*, M9 vs. *P. distasonis*, M9 vs. *B. uniformis*, M9 vs. *B. cellulosilyticus*, M9 vs. *B. fragilis*, M9 vs. *P. dorei*, M9 vs. *B. stercoris*, and M9 vs. *B. longum* groups, and it was significantly upregulated in the M9 vs. *E. coli* group. L-serine not only participates in protein synthesis but also acts as a precursor of nucleotides, amino acids, and phospholipids [44]. L-serine can be converted to pyruvate and used for central metabolism, energy production, and gluconeogenesis [45]. Some studies have shown that serine starvation, which is an intracellular metabolic signal, can activate biofilm formation. The consumption of serine in the *Bacillus subtilis* biofilm has shown that excessive consumption of serine can cause specific serine codons to pause during translation, resulting in a decrease in the translation rate of sinR (a barrier protein encoding biofilm matrix genes) [45]. Three different l-serine deaminases exist in *E. coli* and accelerate the consumption rate of l-serine [46]. The content of l-serine in the M9 biofilm and the other eight mono-species biofilms was significantly higher than that in the *E. coli* biofilm. In addition, L-serine chemoreceptors, i.e., *Tsr* and *LsrB*, can interact in the periplasm to improve the sensing of AI-2 [47].

Purine metabolism was significantly influenced by M9 (Figure 7 and Figure S2). Purine metabolism is crucial for cellular function and is a key pathway that affects DNA and RNA synthesis. Two common DEGs (guanosine and hypoxanthine) were involved in purine metabolism in the present study. Hypoxanthine was significantly co-upregulated in the nine groups (Figure 6). Guanosine was significantly downregulated in the M9 vs. *B. ovatus*, M9 vs. *P. distasonis*, M9 vs. *B. uniformis*, M9 vs. *B. cellulosilyticus*, M9 vs. *B. fragilis*, M9 vs. *P. dorei*, and M9 vs. *B. stercoris* groups, and it was significantly upregulated in the M9 vs. *E. coli* and M9 vs. *B. longum* groups (Figure 6). Guanosine can be catabolized to guanine by purine nucleoside phosphorylase (PNP) and then converted to guanosine 5′-monophosphate (GMP) by hypoxanthine-guanine phosphoribosyl transferase (HGPRT). Hypoxanthine can be catabolized to inosine monophosphate (IMP) by HGPRT and then converted into adenosine monophosphate (AMP) or GMP [48]. Cyclic di-AMP, cAMP, and cyclic di-GMP are the most common secondary messengers that participate in bacterial signal transduction [49]. Secondary messengers are an important mechanism by which cells perceive various environments and respond to internal stimulations, with a notable impact on the formation and development of biofilms. Furthermore, secondary messengers are involved in all stages of biofilm formation and promote the production of EPS including extracellular DNA, protein adhesins, and extracellular polysaccharides [50].

## 5. Conclusions

In this study, the key genes and metabolites that affect M9 biofilm formation were determined using transcriptomes and metabolomes. The results showed that there were 740 common DEGs and four DAMs among all nine groups, which were considered as key genes and metabolites to form the M9 biofilm. Five pathways related to bacterial motility, cellular communication, and signal transduction were enhanced in the M9 biofilm, including biofilm formation—*Escherichia coli*, the two-component system, quorum sensing, ABC transporters, flagellar assembly, and bacterial chemotaxis. Compared with the mono-species biofilms, more peptides/amino acids and derivatives were produced, and purine metabolism was significantly improved in the M9 biofilm. The combined analysis revealed 29 common DEGs highly correlated with four common DAMs, which were coenriched in five amino acids and purine metabolism pathways. This study identified 740 key genes and four key metabolites that can affect M9 biofilm formation using omics technologies, but more research is needed to elucidate the M9 biofilm formation mechanisms and how these key genes and metabolites regulate interactions in the M9 biofilm.

## Figures and Tables

**Table 1:** Specific primers of selected DEGs.

Gene	Forword Primer	Reverse Primer
*gadC*	CGGAAATCGCCTCCTGGATT	GCGAAATGACCAGCGTTACC
*pdeR*	GTCATTGGGCAAAGCGTGTT	TTGCGAAACAGAAACAGCCG
*qseC*	ACCCAACGTCTTAGTCTGCG	TTAACCGCTTGGCAAACAGC
*baeR*	TCGTCTGCTGAAAACGCTCT	ATGGTGCGGTCGGTTACTAC
*csrA*	CCGTGACAGTTTTAGGGGTA	AACTGGACTGCTGGGATTTT
*rcsB*	TATGAACAACAACCCGGCGA	CCGTAACCACCAGCACTGAT
*barA*	ACCTGAAACTTATCGGCGCA	GTTTGTTGCTGATGCGGGAG
*etk*	ACAGAATATCGCTCGCCAGG	CAAGAACGGCTTTGGCTTCC
*gfcE*	CAGAACAAACTGCTGCACCC	GGTCCATTTTCAGCGTGCTC
*cheW*	GTAACACGGATTGCGAACAC	ACATCCACCTGGCTGAACTT
*flgJ*	GGAAGGGATGTTCGTGCAGA	CTGGCAATGGTTGTTCTGGC
*fliR*	TATGCCCGTTTTAGCGCGTA	CAGCATCAGCCCGTTAAGGA
*bcsA*	AGGACAGCTACCCGGAAGAT	ACCAGAGAAGTCAGCACACG
*rbsB*	TGCTGATTAACCCGACCGAC	AGAAGCAATGTGGCTCACCA
16S	GACTACCAGGGTATCTAATC	GTATTACCGCGGCTGCTGGCA

**Table 2:** Overview of RNA-seq of mono-species and M9 biofilms.

Sample	Clean Data	Effective	Q20	Q30
*B. ovatus*	2.81 G	98.67%	98.12%	94.34%
*P. distasonis*	2.15 G	96.06%	96.61%	91.25%
*B. uniformis*	2.03 G	98.74%	98.01%	94.44%
*B. cellulosilyticus*	2.00 G	99.17%	98.39%	94.90%
*E. coli*	2.06 G	98.02%	98.42%	95.40%
*B. fragilis*	2.32 G	93.33%	97.21%	93.17%
*P. dorie*	2.68 G	95.42%	98.10%	94.30%
*B. stercoris*	2.72 G	98.54%	98.07%	94.23%
*B. longum*	2.20 G	99.21%	97.97%	94.42%
M9	18.45 G	98.95%	98.12%	94.48%
Total	39.42 G			

Q20: recognition error rate of bases ≤ 1%; Q30: recognition error rate of bases ≤ 0.1%.

## Data Availability

The original contributions presented in this study are included in this article/Supplementary Material. Further inquiries can be directed to the corresponding authors.

## Supplementary Materials

The following supporting information can be downloaded at: https://www.mdpi.com/article/10.3390/microorganisms13020234/s1, Figure S1: The KEGG enrichment analysis of DEGs. (A–I) Top 10 KEGG enrichment bubble plots of the M9 vs. *B. ovatus*, M9 vs. *P. distasonis*, M9 vs. *B. uniformis*, M9 vs. *B. cellulosilyticus*, M9 vs. *E. coli*, M9 vs. *B. fragilis*, M9 vs. *P. dorei*, M9 vs. *B. stercoris*, and M9 vs. *B. longum* groups. (J) Subcategories of the 86 KEGG pathways. Figure S2: The KEGG enrichment analysis of DAMs. (A–I) Top 10 KEGG enrichment bubble plots of the M9 vs. *B. ovatus*, M9 vs. *P. distasonis*, M9 vs. *B. uniformis*, M9 vs. *B. cellulosilyticus*, M9 vs. *E. coli*, M9 vs. *B. fragilis*, M9 vs. *P. dorei*, M9 vs. *B. stercoris*, and M9 vs. *B. longum* groups. (J) Subcategories of the 36 KEGG pathways. Table S1: Details of the modified YCFA medium (1 L).

## Abbreviations

M9 biofilm	nine-gut-bacteria-formed multi-species biofilm on wheat fibers
DEGs	differentially expressed genes
DAMs	differentially accumulated metabolites
AI-2	autoinducer 2
YCFA	yeast extract, casitone, and fatty acid
NCBI	National Center for Biotechnology Information
STAR	Spliced Transcripts Alignment to a Reference
KEGG	Kyoto Encyclopedia of Genes and Genomes
UHPLC-MS	ultra-performance liquid chromatography–mass spectrometry
ABC transporter	ATP-binding cassette transporter
cAMP	cyclic adenosine 3′5′ monophosphate
CRP	cyclic adenosine 3′5′ monophosphate receptor protein
Csr	carbon storage regulator
PNP	purine nucleoside phosphorylase
GMP	guanosine 5′-monophosphate
HGPRT	hypoxanthine-guanine phosphoribosyl transferase
IMP	inosine monophosphate
AMP	adenosine monophosphate
