# Supplementary material for: Combined Analysis of Transcriptomes and Metabolomes Reveals Key Genes and Substances That Affect the Formation of a Multi-Species Biofilm by Nine Gut Bacteria
Source: Microorganisms. 2025 Jan 22;13(2):234. doi: 10.3390/microorganisms13020234 (PMC11857192; doi:10.3390/microorganisms13020234)
Supplement: Supplementary file 1 [file microorganisms-13-00234-s001.zip › microorganisms-3426432-supplementary.pdf]

microorganisms

Supplementary Materials for:

Combined Analysis of Transcriptome and Metabolome Reveals Key Genes and Substances That Affect Formation of Multi-Species Biofilm by Nine Gut Bacteria

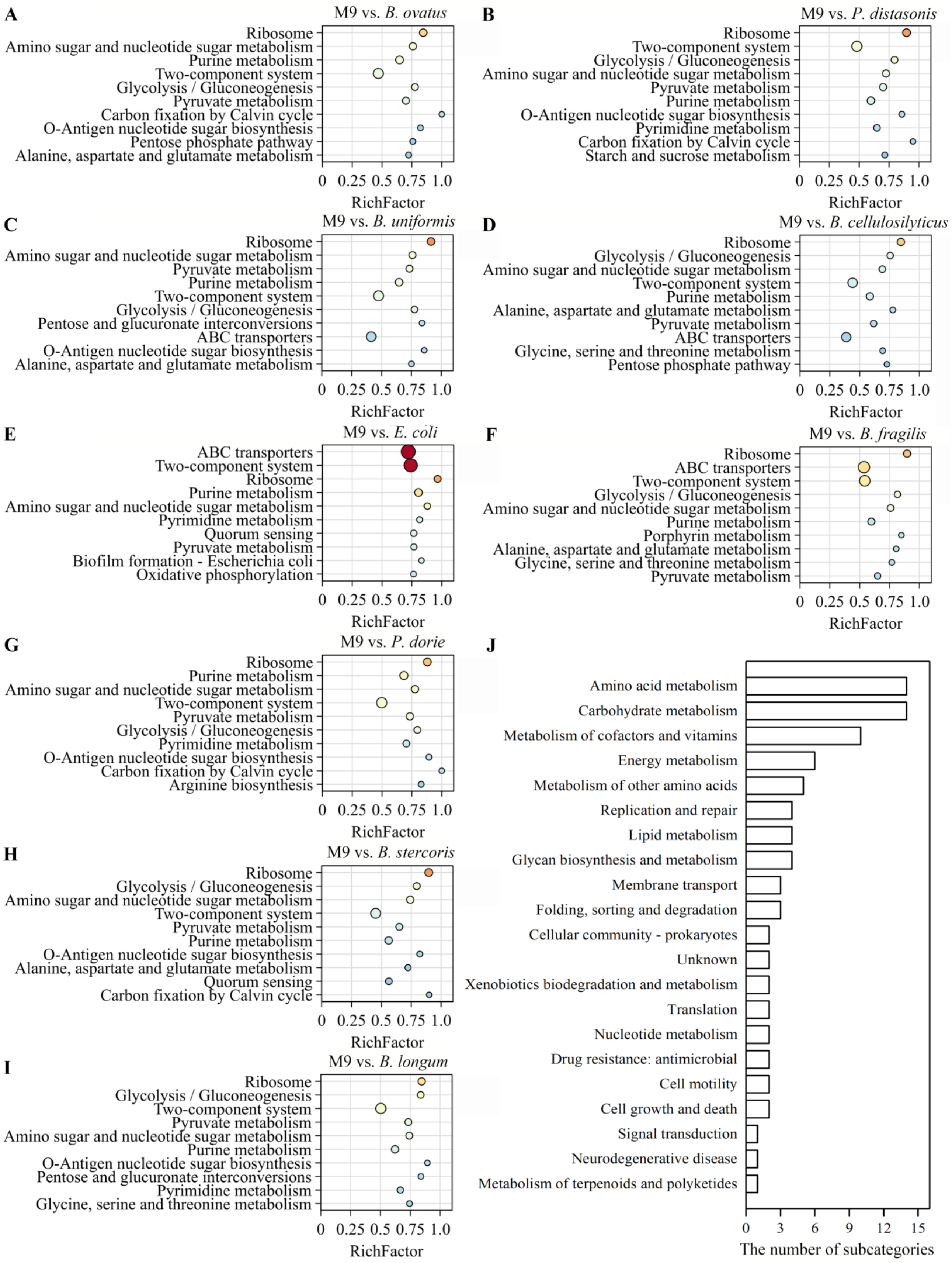

Figure S1: The KEGG enrichment analysis of DEGs. (A-I) Top 10 KEGG enrichment bubble plots of M9 vs *B. ovatus*, M9 vs *P. distasonis*, M9 vs *B. uniformis*, M9 vs *B. cellulosilyticus*, M9 vs *E. coli*, M9 vs *B. fragilis*, M9 vs *P. dorei*, M9 vs *B. stercoris*, and M9 vs *B. longum* groups. (J) Subcategories of the 86 KEGG pathways.

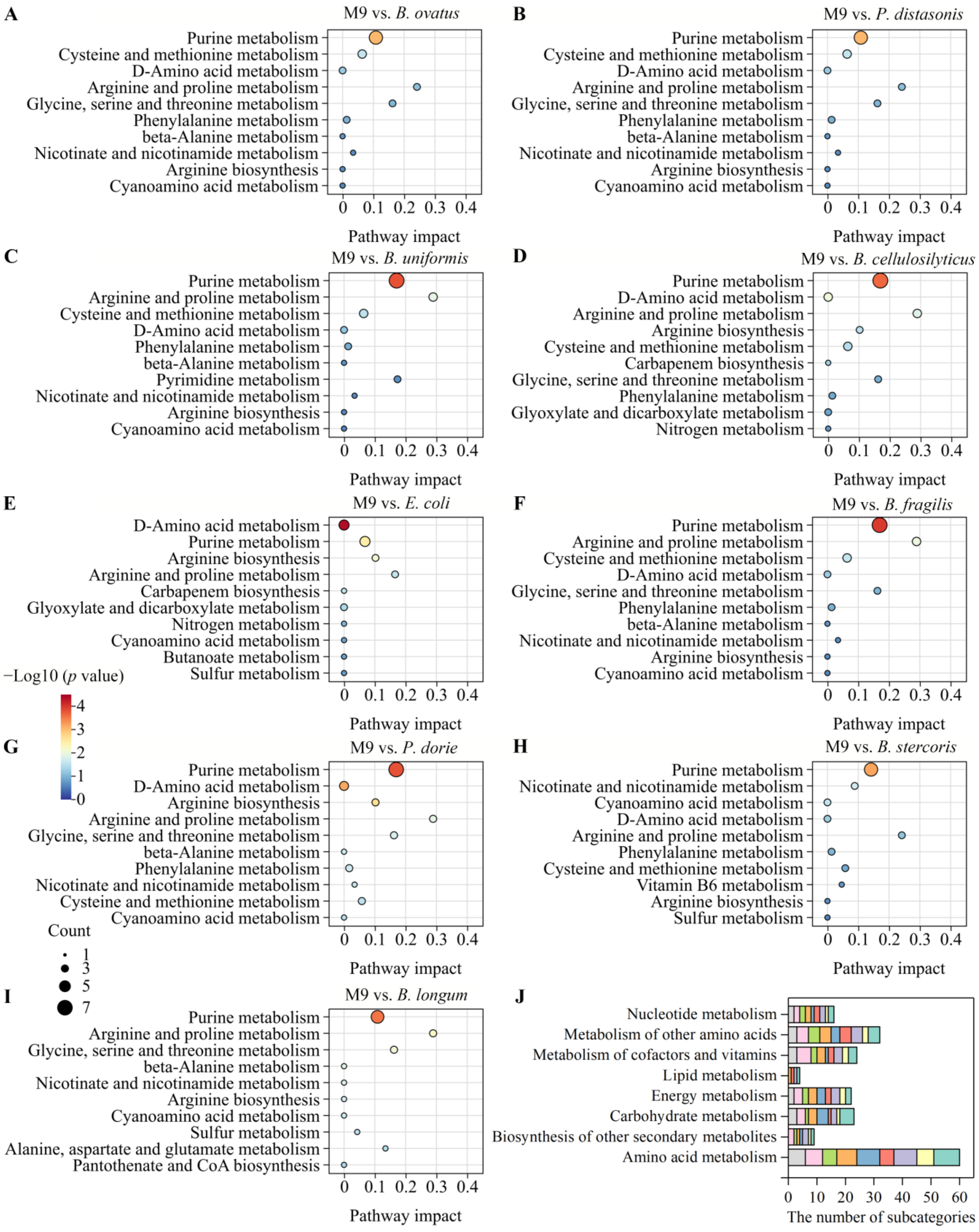

Figure S2: The KEGG enrichment analysis of DAMs. (A-I) Top 10 KEGG enrichment bubble plots of M9 vs *B. ovatus*, M9 vs *P. distasonis*, M9 vs *B. uniformis*, M9 vs *B. cellulosilyticus*, M9 vs *E. coli*, M9 vs *B. fragilis*, M9 vs *P. dorei*, M9 vs *B. stercoris*, and M9 vs *B. longum* groups. (J) Subcategories of the 36 KEGG pathways.

Table S1: Details of modified YCFA medium (1L).

Formulation	Content	Formulation	Content

Casein peptone	10 g	Cellobiose	2g
Tryptone	10 g	Maltose	2 g
Yeast extract	5 g	Biotin	0.01 mg
Sodium acetate	5 g	Vitamin B12	0.01 mg
Lactose·H2O	5 g	p-Aminobenzoic acid	0.03 mg
NaHCO3	4 g	Folic acid	0.05 mg
(NH4)2SO4	1.8 g	Pyridoxine-HCl	0.15 mg
KH2PO4	0.45 g	Vitamin B1	0.05 mg
NaCl	0.9 g	Riboflavin	0.05 mg
MgSO4 × 7 H20	0.045 g	D-Ca-pantothenate	5 mg
CaCl2	0.09 g	Hemin chloride	1%
L-Cysteine-HCl	1.445 g	Clarify bovine rumen fluid	0.5%
Glucose	2g		
